# Identifying subtypes of HIV/AIDS-related symptoms in China using latent profile analysis and symptom networks

**DOI:** 10.1038/s41598-022-17720-z

**Published:** 2022-08-02

**Authors:** Zhongfang Yang, Zheng Zhu, Huan Wen, Shuyu Han, Lin Zhang, Yanfen Fu, Yan Hu, Bei Wu

**Affiliations:** 1 Shanghai Institute of Infectious Disease and Biosecurity, School of Nursing, Fudan University, Shanghai, China; 2 School of Nursing, Fudan University, 305 Fenglin Rd., Shanghai, China; 3 Fudan University Centre for Evidence-Based Nursing: A Joanna Briggs Institute Centre of Excellence, Fudan University, Shanghai, China; 4 NYU Rory Meyers College of Nursing, New York University, New York City, NY USA; 5 School of Public Health, Fudan University, Shanghai, China; 6 School of Nursing, Peking University, Beijing, China; 7 Shanghai Public Health Clinical Center, Shanghai, China; 8 School of Nursing, Dali University, Dali, Yunnan China

**Keywords:** Quality of life, HIV infections, Comorbidities

## Abstract

The identification of subgroups of people living with HIV in China based on the severity of symptom clusters and individual symptoms is crucial to determine group-specific symptom management strategies. Participants reported 27 highly prevalent HIV/AIDS-related symptoms. Latent profile analysis based on symptom severity was used to identify person-centered subtypes of HIV/AIDS-related symptoms. Symptom networks were compared among subgroups identified by latent profile analysis. A total of 2927 eligible people living with HIV (PWH) were included in the analysis. Five profiles were identified: “Profile 1: all low symptom severity” (n_2_ = 2094, 71.54%), “Profile 2: medium symptom severity with syndemic conditions” (n_3_ = 109, 3.72%), “Profile 3: medium symptom severity with low functional status” (n_1_ = 165, 5.64%), “Profile 4: medium symptom severity in transitional period” (n_4_ = 448, 15.31%), and “Profile 5: all high symptom severity” (n_5_ = 111, 3.79%). Except for Profile 1 and Profile 5, the symptom severity was similar among the other three profiles. Profiles 1 (2.09 ± 0.52) and 4 (2.44 ± 0.66) had the smallest ∑s values, and Profiles 2 (4.38 ± 1.40) and 5 (4.39 ± 1.22) had the largest ∑s values. Our study demonstrates the need for health care professionals to provide PWH with group-specific symptom management interventions based on five profiles to improve their physical and psychological well-being. Future studies should be conducted in different contexts using different symptom checklists to further validate our results.

## Introduction

Antiretroviral therapy (ART), with its wide availability, has transformed HIV/AIDS from a fatal acute disease to a manageable chronic condition^1–4^. Despite the use of effective ART, people with HIV (PWH) face increasing challenges related to the physical and psychological symptoms caused by HIV infection, opportunistic infections, the side effects of ART, chronic comorbidities, aging, and social discrimination^5–8^. It is widely reported that PWH experience multiple symptoms simultaneously^9–12^. These symptoms may impact the quality of life, medication adherence, physical and psychological functioning, and social engagement of PWH, which could have a long-term effect on achieving the “90–90–90–90” target^13–15^.

PWH may have a variety of complex symptom manifestations. Common and severe symptoms in PWH include fatigue, sleep disturbance, memory loss, and dizziness. The prevalence of these symptoms may vary across studies and with different study designs^13,14^. In addition, it is likely that PWH experience multiple symptoms simultaneously. Wilson and colleagues found that more than 30% of PWH in the United States experienced an average of seven or more symptoms concurrently^15^. Zhu and colleagues found that the median number of PWH with cooccurring symptoms was nine^12,16^.

The majority of previous studies investigated symptoms separately, which may not reflect the complex real-world situations that PWH experience^16–18^. Zhu and colleagues investigated 1116 PWH in China and found five symptom clusters: cognitive dysfunction, mood disturbance, wasting syndrome, dizziness/headache, and skin-muscle-joint disorder^17^. Moens and colleagues conducted a cross-sectional study in 217 PWH in South Africa and Uganda and summarized five symptom clusters: dermatological, generalized anxiety and elimination, social and image, persistently present, and gastrointestinal-related^18^. A systematic review included thirteen studies exploring HIV/AIDS symptom clusters and found five common symptom clusters: sad/depressed/loss of interest/nervous/anxious/worrying, difficulty sleeping/problems with having sex/fatigue, fever/chills/sweat/nausea/vomiting/loss of appetite, numbness/muscle ache/joint pain, and dizziness/headache^16^.

Identifying symptom clusters is a commonly used approach for dimension reduction to simplify complex relationships among symptoms in real-world clinical practice. This method can not only enhance the efficiency of symptom management strategies but also prevent the occurrence of symptoms in the same cluster^19^. However, many reviews have noted that the combinations of symptoms in clusters may vary due to the selection of symptoms included in the analysis, the statistical methods used, and other covariates^20,21^. Whether the dimension reduction approach to exploring symptom clusters suits today’s clinical practice with large amounts of big data continues to be debated^22^.

Categorized symptoms give only a broad picture of which HIV/AIDS-related symptoms share the same cooccurring mechanisms^23,24^. It is still unclear whether demographic and health-related characteristics differ among PWH with different symptom subgroups. Few studies have focused on differentiating profiles and symptom networks of clinical subtypes of HIV/AIDS-related symptoms rather than creating symptom clusters. The identification of subgroups of PWH based on the severity of symptom clusters and individual symptoms is crucial to determine group-specific management strategies^25^.

Latent profile analysis (LPA), as a patient-centered analytic methodology, is used to identify subgroups or classes of individuals within a sample or population who share comparable characteristics or symptom experiences. Different from variable-centered approaches such as factor analysis, LPA can distinguish salient characteristics and evaluate how patient outcomes differ by profiles. In addition, LPA fit indices are assessed before choosing the final LPA model, which is more objective than patient-centered analytic methodology^26^.

In this study, symptom networks were used based on the LPA results. A symptom network is defined as a new paradigm for analyzing complex interconnectedness among multiple symptoms. Although the concept of symptom networks originated in psychopathology, over the past three years the paradigm has been used to capture the complex relationships between symptoms of various chronic diseases^22^. Symptom networks explore and visualize the internal mechanisms of symptoms in a given population, which can help researchers not only identify the potential causality of symptoms but also explore core symptoms from a mechanism perspective. Indices of symptom networks, such as centrality and density, have been reported in previous studies as more sensitive indicators than symptom severity and occurrence^27^. Based on network theory, using symptom networks can further differentiate the profiles and provide additional data that patient-centered analytic methodology could not find. Using LPA and symptom networks together may have more clinical implications and could lead to the development of a more precise and individualized intervention^28,29^.

Therefore, the objectives of this study were to 1) generate subgroups of HIV/AIDS-related symptoms by latent profile analysis (LPA) and 2) determine whether the subgroups differ in demographic and health-related characteristics and indicators for symptom networks.

## Methods

### Study design

This study used data from the HIV-related Symptoms Monitoring Survey (HSMS). The HSMS is a cross-sectional dataset collected by our team that includes PWH from 11 cities in eastern (Shanghai City), central (Changsha in Hunan Province), and southwestern (Ruili, Tengchong, Kunming, Longxing, Changning, Baoshan, Linchang, and Longchuan in Yunnan Province and Nanning in Guangxi Province) China from 2017 to 2019. More information regarding the HSMS can be found elsewhere^5^. Ethics approval was obtained from the institutional review board of the School of Nursing, Fudan University (IRB#TYSA2016-3-1). This research was performed in accordance with the Declaration of Helsinki. Written informed consent was obtained from participants before data collection.

### Sample

Participants were included in the study if they were (1) HIV positive and (2) aged ≥ 18 years and over. PWH who did not complete a self-reported symptom checklist or (3) were diagnosed with severe neurocognitive disorders were excluded from the study. From 2017 to 2019, we recruited 3017 participants through a convenience sample from 11 hospitals in 11 cities as mentioned in the study design, which are responsible for HIV/AIDS-related treatment and care in these areas. Ninety participants were excluded due to missing data. As a result, a total of 2927 eligible PWH were included in the analysis.

### Measures

#### Sociodemographic and clinical data

Demographic, socioeconomic, and clinical data were collected from a self-administered questionnaire. Demographic variables included age (continuous), sex (male and female), and ethnicity (Han and minority). Socioeconomic variables included marital status (married, single, and otherwise), employment status (employed and otherwise), educational attainment (middle school or below, high school or equivalent, bachelor’s degree or equivalent, and master’s degree or above), and primary caregiver (myself, family members, and others). Clinical variables included years since HIV diagnosis (in years, continuous), ART use (yes or no), ART use duration (in years, continuous), latest CD4+T-cell count (continuous), and comorbidities (yes or no). All sociodemographic and clinical data were confirmed by medical records.

#### Self-reported symptoms

The HIV/AIDS Self-reported Symptom Checklist (HSSC) was used to evaluate the severity of 27 highly prevalent HIV/AIDS-related symptoms^16^. The twenty-seven symptoms included in the HSSC were categorized into 5 symptom clusters (wasting syndrome, dizziness/headache, cognitive function, skin-muscle-joint disorder, and mood disturbance) and 7 individual symptoms (fatigue, sleep disturbance, cough, hair loss, blurred vision, low sex drive, and lipodystrophy). The responses ranged from not at all (0) to severe (3). The total score was determined by summing the scores of these 27 items (ranging from 0 to 81). The total scores for the wasting syndrome cluster, dizziness/headache cluster, cognitive function cluster, skin-muscle-joint disorder cluster, and mood disturbance cluster were 15, 6, 15, 12, and 12, respectively. The HSSC had good expert validity (content validity index = 0.918) and internal consistency (Cronbach’s α = 0.961).

#### Basic activities of daily living

The ability to perform basic activities of daily living was assessed by the Barthel Index (BI)^30^. The BI is a widely used measure for evaluating the ability to perform basic activities of daily living, such as bathing, dressing, and grooming. The BI contains 10 items, and the total score ranges from 0 to 100. A higher score indicates a higher ability to perform basic activities of daily living. The measure showed good internal consistency in our sample (Cronbach’s α = 0.941).

#### Medication adherence

Patient-reported medication adherence was measured by one question: “How often did you forget to take your medication in the last 7 days?” The response ranged from never (1) to all the time (5).

#### Discrimination perceived by PWH

The discrimination perceived by PWH was assessed by the Expanded Everyday Discrimination Scale^31^. This measure includes 10 items and describes different scenarios in day-to-day life in which PWH may perceive discrimination. The total score ranges from 10 to 40. A higher score indicates a lower level of perceived discrimination. The measure showed good internal consistency in our sample (Cronbach’s α = 0.913).

#### Self-reported health condition, quality of life, and self-management capacity

Self-reported health condition, quality of life, and self-management capacity were measured by the questions “How do you rate your overall health condition?”, “How do you rate your quality of life?”, and “How do you rate your self-management capacity for HIV/AIDS?” Responses for these variables ranged from very good (1) to very bad (5).

### Statistical analysis

Mplus 8.1 was used to perform LPA to identify person-centered subtypes of HIV/AIDS-related symptoms. LPA was conducted based on the severity of symptom clusters (wasting syndrome, dizziness/headache, cognitive function, skin-muscle-joint disorder, and mood disturbance) and individual symptoms (fatigue, sleep disturbance, cough, hair loss, blurred vision, low sex drive, and lipodystrophy). We calculated the total severity score for each cluster (dizziness/headache, cognitive dysfunction, skin muscle joint disorder, wasting syndrome, mood disturbance). For 7 symptoms that could not be categorized into clusters, we used Likert items to assess the severity of each symptom. We used LPA due to the severity scores being continuous response variables, whereas latent class analysis (LCA) was used for categorical variables. The number of classes was determined by comparing the Bayesian information criterion (BIC), Akaike information criterion (AIC), sample-size-adjusted BIC (ABIC), Lo-Mendell-Rubin likelihood ratio test (LMR), bootstrapped likelihood ratio test (BLRT) and entropy of each model. Smaller AIC, BIC and ABIC values indicate a better model fit. P values higher than 0.05 for the LMR and BLRT indicated that the k−1 model was rejected and that the k model was supported. Theoretical interpretability was also taken into consideration. We plotted the conditional probabilities of symptom severity for each of the classes. The code for conducting LPA in Mplus is shown in Supplementary file 1.

After identifying the classes, we analyzed the differences in the sociodemographic and clinical data, basic activities of daily living, medication adherence, perceived discrimination, self-reported health condition, quality of life, and self-management capacity by using the chi-square test and one-way analysis of variance (ANOVA) with post hoc tests (Fisher’s least significant difference). Tamhane’s T2 multiple comparison test was used if the variances were heterogeneous. Multinomial logistic regression analysis of the five profiles was further conducted. The Nagelkerke R^2^ and χ^2^ were used as indicators for model fitness. We considered a two-tailed *P* < 0.05 to indicate statistical significance in all analyses.

R 4.0.2 and the Qgraph module were used to conduct the network analysis. We used Spearman correlations to assess the relationships (edges) between pairs of symptoms (nodes) in the full sample and subgroups. The Fruchterman-Reingold (FR) algorithm and spring layout were used to generate symptom networks^26^. In the FR algorithm, the node (symptom) with the strongest centrality was placed in the center of the network, and nodes with similar characteristics were placed more closely. Covariates were selected from bivariate analysis results and included age (continuous), sex (male = 1, female = 2), ethnicity (Han = 1, minority = 2), educational attainment (middle school or below = 1, high school or above = 2), employment (employed = 1, otherwise = 2), marital status (married = 1, otherwise = 2), primary caregiver (myself = 1, otherwise = 2), having ART (yes = 1, no = 2), years of having ART (continuous), having comorbidities (yes = 1, no = 2), lgCD4 (continuous), medication adherence (continuous), self-management capacity (continuous), and perceived discrimination (continuous).

We used three centrality indices (strength, betweenness, and closeness) to identify the most central symptoms^32,33^. Strength is a measure of network connectivity. The greater the strength is, the higher the probability that the symptom will cooccur with other symptoms. Betweenness quantifies the number of times a node acts as a bridge along the shortest path between two nodes. A node with higher betweenness centrality has more influence on the network. Closeness represents the average farness (inverse distance) from one symptom to all other nodes. The greater the value of closeness is, the shorter the path. For a contemporaneous network, strength is used as the major indicator among the three indices. We used the absolute value of all Spearman coefficients (∑s) to indicate the density of symptom network interconnections.

### Ethical approval

Ethics approval was obtained from the institutional review board of the School of Nursing, Fudan University (IRB#TYSA2016-3-1).

## Results

### Sample characteristics

Overall, the mean age of the participants was 42.26 years, ranging from 10 to 87 years old. The majority of participants were male (63.7%), of Han ethnicity (74.8%), married (55.3%), not employed (59.7%), and had a middle school or below educational attainment (64.7%). The average years since HIV diagnosis were 5.66 years (0–36 years). Among all participants, 96.7% received ART. The average duration of ART use was 4.49 ± 3.83 years (0–24 years). The average latest CD4+T-cell count was 411.37 ± 264.55 cell/mm^3^ (1.00 ~ 2125.00 cell/mm^3^). Approximately one-third of the participants (31.2%) had comorbidities.

### Symptom subgroups identified by LPA

LPA identified five subgroups of participants based on the rating of symptom severity. Table 1 shows the results of latent profile model fit indices. Although the six-profile model had the lowest AIC (90,046.269), BIC (90,578.643), and aBIC (90,295.857) values, the BLRT and LMR-LRT tests indicated that the five-profile model was more appropriate than the six-profile model. The five-profile model also showed the highest entropy value (0.955). In addition, five latent profiles were selected based on the clinical significance and results from our previous studies^5,17^.

**Table 1:** Latent profile model fit indices (n = 2927).

Model	Loglikelihood	AIC	BIC	aBIC	Entropy	LMR *p*-value	LMR meaning	BLRT p-value	BLRT meaning
1	− 51,603.287	103,254.574	103,398.136	103,321.879	–	–		–	
2	− 47,609.965	95,293.929	95,515.253	95,397.691	0.953	0.001	2 > 1	< 0.0001	2 > 1
3	− 46,397.255	92,894.510	93,193.597	93,034.728	0.932	0.006	3 > 2	< 0.0001	3 > 2
4	− 45,710.175	91,546.350	91,923.199	91,723.025	0.953	0.001	4 > 3	< 0.0001	4 > 3
5	− 45,375.132	90,902.263	91,356.875	91,115.395	0.955	0.006	5 > 4	< 0.0001	5 > 4
6	− 44,934.135	90,046.269	90,578.643	90,295.857	0.945	0.135	6 < 5	< 0.0001	6 > 5

**Figure 1:**
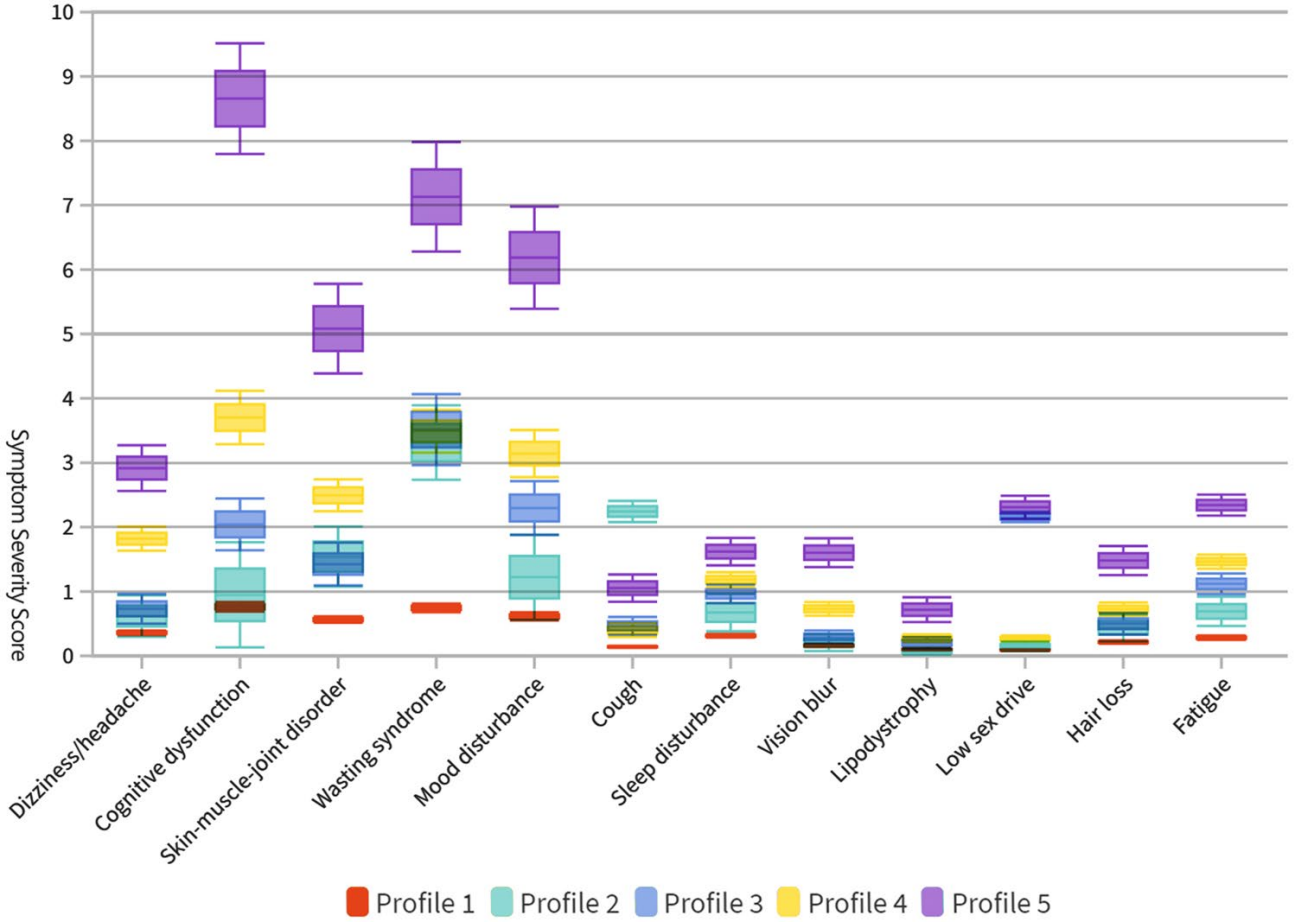
Symptom severity score of 5 latent profiles for 5 symptom clusters and 7 symptoms (n = 2927).

Figure 1 shows symptom severity score plots for the five-profile model. The purple line (Profile 2) shows the subgroup with medium–high symptom severity in 5 symptom clusters and fatigue (3.72%, n = 109). The bluish-green line (Profile 3) shows the subgroup with a medium–high severity of symptoms, including cognitive function, sex function, and sleep function (5.64%, n = 165). The blue line (Profile 4) shows the subgroup with a medium–high severity of symptoms related to opportunistic infections, such as wasting syndrome and cough (15.31%, n = 448).

### Differences in demographic and health-related characteristics among subgroups

Table 2 shows the demographic and health-related characteristic differences among the 5 subgroups identified by LPA. Except for medication adherence (*P* = 0.321), there were significant differences in all demographic and health-related characteristics among the 5 subgroups (*P* < 0.05). Table 3 shows the multinomial logistic regression analysis of the five profiles. Profile 5 was chosen as the reference group. Being male (OR = 2.103–2.729), longer duration of using ART (OR = 0.834–0.851), having comorbidity (OR = 0.278), lower level of basic activity of daily living (OR = 1.033) and self-reported health condition (OR = 0.327–0.558), and higher level of perceived discrimination (OR = 1.073 = 1.234) were associated with higher likelihood in profile 5.

**Table 2:** Demographic and health-related characteristics difference among subgroups.

Characteristics	M ± SD or n (%)						*P* value
	Total (N = 2927)	Profile 1 (n = 2094)	Profile 2 (n = 109)	Profile 3 (n = 165)	Profile 4 (n = 448)	Profile 5 (n = 111)	
Age	42.26 ± 12.21	41.85 ± 11.95	44.50 ± 12.49	43.22 ± 13.22	43.02 ± 12.39	43.32 ± 14.10	0.046
**Sex**							
Male	1864 (63.68)	1340 (63.99)	72 (66.06)	124 (75.15)	263 (58.71)	65 (58.56)	0.003
Female	1063 (36.32)	754 (36.01)	37 (33.94)	41 (24.85)	185 (41.29)	46 (41.44)	
**Ethnicity**							
Han	2188 (74.75)	1529 (73.02)	88 (90.73)	130 (78.79)	353 (78.79)	88 (79.28)	0.018
Minority	739 (25.25)	565 (26.98)	21 (19.27)	35 (21.21)	95 (21.21)	23 (20.72)	
**Employment status**							
Employed	1180 (40.31)	924 (44.13)	26 (23.85)	54 (32.73)	151 (33.71)	25 (22.52)	< 0.0001
Otherwise	1747 (59.69)	1170 (55.87)	83 (76.15)	111 (67.27)	297 (66.29)	86 (77.48)	
**Primary caregiver**							
Myself	1100 (37.58)	834 (39.83)	32 (23.96)	61 (36.70)	150 (33.48)	23 (20.72)	< 0.0001
Family members	1596 (54.53)	1101 (52.58)	69 (63.30)	90 (54.55)	252 (56.25)	84 (75.68)	
Otherwise	231 (7.89)	159 (7.59)	8 (7.34)	14 (8.48)	46 (10.27)	4 (3.60)	
Years since HIV diagnosis	5.66 ± 4.85	5.80 ± 4.85	4.79 ± 5.06	4.93 ± 4.71	5.36 ± 4.62	6.17 ± 5.64	0.017
**Use of ART**							
Yes	2829 (96.65)	2041 (97.47)	97 (88.99)	157 (95.15)	428 (95.54)	106 (95.50)	< 0.0001
No	98 (3.35)	53 (2.53)	12 (11.01)	8 (4.85)	20 (4.46)	5 (4.50)	
Duration of using ART	4.49 ± 3.83	4.56 ± 3.77	3.77 ± 3.52	3.78 ± 3.86	4.46 ± 3.90	5.11 ± 4.81	0.010
CD4 + T cell count	411.37 ± 264.55	429.38 ± 298.27	337.59 ± 264.79	340.75 ± 220.21	341.95 ± 228.02	353.20 ± 306.52	< 0.0001
**Comorbidities**							
Yes	912 (31.16)	536 (25.60)	33 (33.28)	82 (49.70)	199 (44.42)	62 (55.86)	< 0.0001
No	2015 (68.84)	1558 (74.40)	76 (69.72)	83 (50.30)	249 (55.58)	49 (44.14)	
Basic activity of daily living	96.91 ± 12.28	98.94 ± 6.97	92.78 ± 17.61	91.40 ± 18.20	93.01 ± 19.10	86.79 ± 22.33	< 0.0001
Medication adherence	0.88 ± 0.75	0.89 ± 0.74	0.87 ± 0.87	0.81 ± 0.76	0.85 ± 0.76	1.01 ± 0.69	0.321
Self-reported health condition	3.19 ± 1.11	2.91 ± 1.05	3.80 ± 0.93	3.86 ± 0.93	3.84 ± 0.93	4.22 ± 0.82	< 0.0001
Self-reported quality of life	3.21 ± 1.04	3.00 ± 1.04	3.53 ± 0.98	3.73 ± 0.78	3.73 ± 0.83	4.02 ± 0.85	< 0.0001
Self-management capacity	2.81 ± 1.09	2.65 ± 1.04	3.17 ± 1.07	3.15 ± 1.05	3.18 ± 1.10	3.47 ± 1.26	< 0.0001
Perceived discrimination	37.70 ± 4.14	38.15 ± 3.73	38.48 ± 3.21	36.93 ± 5.15	36.39 ± 4.68	33.79 ± 5.65	< 0.0001

**Table 3:** Multnomial logistic regression analysis of five profiles.

Variable	OR (95% CI)			
	Profile 1 versus Profile 5	Profile 2 versus Profile 5	Profile 3 versus Profile 5	Profile 4 versus Profile 5
Age	1.018 (0.993, 1.043)	1.034 (1.002, 1.068)	1.011 (0.982, 1.040)	1.009 (0.984, 1.035)
Male (compared to female)	2.103 (1.180, 3.747)*	2.035 (0.929, 4.456)	2.729 (1.357, 5.487)**	1.423 (0.782, 2.593)
Han ethnicity (compared to minority)	0.777 (0.424, 1.423)	1.084 (0.464, 2.535)	1.055 (0.511, 2.176)	1.196 (0.634, 2.255)
Employed (compared to otherwise)	1.493 (0.794, 2.807)	0.710 (0.289, 1.744)	1.429 (0.680, 3.001)	1.406 (0.731, 2.704)
Primary caregiver: myself (compared to otherwise)	1.854 (0.985, 3.489)	1.719 (0.747, 3.960)	2.020 (0.976, 4.183)	1.599 (0.830, 3.079 )
Years since HIV diagnosis	1.051 (0.951, 1.161)	1.036 (0.907, 1.184)	1.039 (0.926, 1.166)	1.000 (0.900, 1.110)
Use ART	0.742 (0.085, 6.480)	–^#^	0.807 (0.065, 9.986)	0.954 (0.098, 9.277)
Duration of using ART	0.851 (0.756, 0.958)**	0.834 (0.705, 0.987)*	0.880 (0.763, 1.013)	0.918 (0.811, 1.039)
Lg CD4 + T cell count	1.176 (0.617, 2.242)	1.369 (0.544, 3.449)	1.017 (0.483, 2.141)	1.511 (0.771, 2.964)
Having Comorbidity	0.374 (0.213, 0.656 )	0.278 (0.121, 0.637)**	0.792 (0.406, 1.546)	0.752 (0.421, 1.344 )
Basic activity of daily living	1.033 (1.014, 1.053)**	1.009 (0.985, 1.033)	1.004 (0.985, 1.023)	1.003 (0.987, 1.020)
Medication adherence	0.954 (0.670, 1.356)	1.178 (0.721, 1.922)	0.995 (0.649, 1.526)	0.896 (0.621, 1.292)
Self-reported health condition	0.327 (0.215, 0.498)**	0.929 (0.530, 1.630)	0.558 (0.342, 0.912)*	0.690 (0.447, 1.065)
Self-reported quality of life	0.771 (0.504, 1.180)	0.719 (0.410, 1.262)	1.093 (0.658, 1.814)	1.006 (0.646, 1.566)
Self-management capacity	1.161 (0.873, 1.543)	0.974 (0.650, 1.460)	1.008 (0.716, 1.419)	0.993 (0.741, 1.333)
Perceived discrimination	1.148 (1.096, 1.204)**	1.234 (1.116, 1.366)**	1.105 (1.041, 1.173)**	1.073 (1.024, 1.125)**

Significant values are in [bold].

Model fitness: Nagelkerke R^2^ = 0.230, χ^2^ = 522.403, *P* < 0.0001.

*P < 0.05, **P < 0.01, ^#^insufficient statistical power in subgroup.

### Symptom networks and centrality indices of subgroups

**Figure 2:**
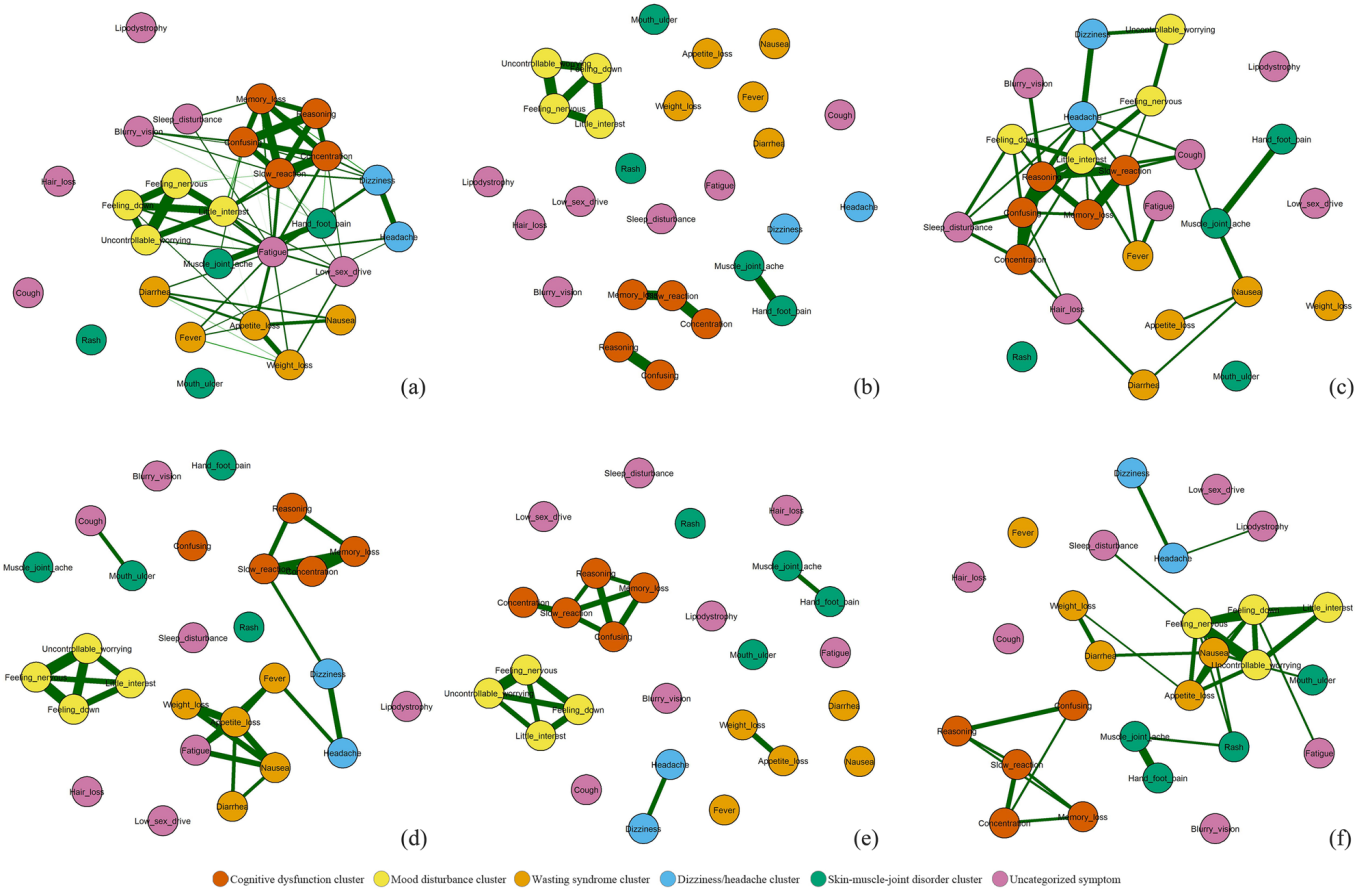
Symptom network in the full sample and 5 subgroups: (**a**) full sample, (**b**) Profile 1, (**c**) Profile 2, (**d**) Profile 3, (**e**) Profile 4, and (**f**) Profile 5. Red nodes represent cognitive dysfunction cluster; yellow nodes represent mood disturbance cluster; orange nodes represent wasting syndrome cluster; green nodes represent skin-muscle-joint disorder cluster; pink nodes represent uncategorized symptoms. In the Fruchterman-Reingold algorithm, the node with the strongest centrality was placed in the center of the network. The thickness of edges corresponds to the strength of association.

Figure 2 shows the symptom networks of the full sample and five subgroups. One-way ANOVA showed that the ∑s was significantly different across the five subgroups (F = 32.441, *P* < 0.0001). The least significant difference test confirmed that differences in the ∑s between Profiles 1 and 4 were not significant (*P* = 0.192). The ∑s of Profiles 2 and 5 were not significantly different (*P* = 0.980). Profiles 1 (2.09 ± 0.52) and 4 (2.44 ± 0.66) had the smallest ∑s values, and Profiles 2 (4.38 ± 1.40) and 5 (4.39 ± 1.22) had the largest ∑s values.

**Figure 3:**
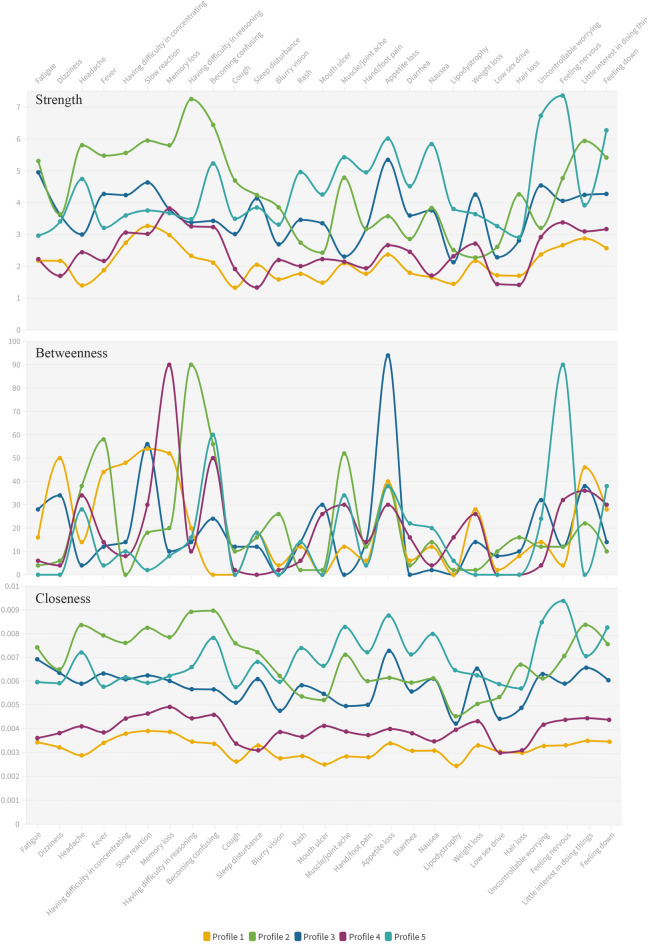
Centrality indices of the networks of the 5 subgroups. Strength is a measure of network connectivity. Betweenness quantifies the number of times a node acts as a bridge along the shortest path between two nodes. Closeness represents the average farness (inverse distance) from one symptom to all other nodes. For a contemporaneous network, strength is used as the major indicator among three indices.

Figure 3 shows the three centrality indices after controlling for covariates in these five subgroups. In the network of Profile 1 (Fig. 2b), slow reaction was the most central symptom based on strength (r_S_ = 3.267), closeness (r_C_ = 0.004), and betweenness (r_S_ = 54.000). In the network of Profile 2 (Fig. 2c), having difficulty reasoning had the largest value for strength (r_S_ = 7.253) and betweenness (r_B_ = 90.000). Becoming confused had the largest value for closeness (r_C_ = 0.009). In the network of Profile 3 (Fig. 2d), appetite loss had the largest values for strength (r_S_ = 5.344), closeness (r_C_ = 0.007), and betweenness (r_S_ = 94.000). In the network of Profile 4 (Fig. 2e), memory loss was the most central symptom across the three centrality indices (r_S_ = 3.818, r_B_ = 90.000, and r_C_ = 0.005). Most of the strength and closeness in the networks of Profiles 2, 3, and 5 were larger than those in Profiles 1 and 4 except memory loss, difficulty reasoning, lipodystrophy, and weight loss.

### Names of the five profiles

Based on the results of LPA and comparing demographic and health-related characteristics and symptom networks, the five profiles were named as follows: “Profile 1: medium symptom severity with low functional status”, “Profile 2: all low symptom severity”, “Profile 3: medium symptom severity with syndemic conditions”, “Profile 4: medium symptom severity in transitional period”, and “Profile 5: all high symptom severity”.

## Discussion

This is the first study to identify five profiles of PWH by conducting LPA and comparing the characteristics and symptom networks of PWH. “Profile 1: all low symptom severity”, “Profile 2: medium symptom severity with syndemic conditions”, “Profile 3: medium symptom severity with low functional status”, “Profile 4: medium symptom severity in transitional period”, and “Profile 5: all high symptom severity”. Except for “Profile 1: all low” and “Profile 5: all high”, the symptom severity was similar in the other three profiles. Profiles 2, 3, and 4 were further differentiated by the comparison of symptom networks and centrality indices.

### Profile 1: all low symptom severity

We found that over 70% of participants were classified in profile 1. PWH in this group not only reported low levels of symptom severity and perceived discrimination but also reported high levels of ADL, medication adherence, quality of life, and self-management capacity. Our results were consistent with previous studies showing that although PWH may have multiple symptoms at same time, the symptom conditions of the majority of PWH may not influence their daily life^34,35^. We found that this proportion in our study was approximately 70% in China.

### Profile 2: medium symptom severity with syndemic conditions

Profile 2 was named “syndemic conditions” due to high density of symptom networks. A high density of symptom networks represented a high probability of having opportunistic infections and comorbidities. Zhu and colleagues conducted a cross-sectional study and found that PWH with a time since HIV-positive diagnosis of 6 months or less or longer than 10 years who had more comorbidities had denser symptom networks than PWH with other times since HIV-positive diagnosis^5^. Schweren’s study identified that symptom network density could be a prognostic indicator of treatment and the consequences of having long-term illness and comorbidities^36^.

Previous studies showed that high symptom network density indicated that symptoms, especially wasting syndrome clusters and cough, were closely related to and might aggravate other symptoms quickly^5,37^. Shkarin and colleagues reported that the pathogen of opportunistic infections could go undetected in the human body, causing potential endogenous infections and thus resulting in complex comorbidities that could lead to severe symptoms^38^. Sandler and Douek evaluated the mechanism of opportunistic infections from the intestinal lumen into systemic circulation and found a key role of endogenous infections in immune activation and disease progression in PWH^39^. Health care professionals may need to pay close attention to PWH in this profile who report a medium level of symptom severity but may have simultaneous infection.

### Profile 3: medium symptom severity with low functional status

Profile 3 was named “low functional status” due to its high severity of functional symptoms such as cognitive function, sleep disturbance, and low sex drive. PWH in Profile 3 reported similar levels of CD4 compared to those in Profile 2 but had a higher level of medication adherence, which indicated that Profile 3 could be the comparison group of Profile 2 if PWH maintain a high level of ART adherence. The high prevalence and severity of cognitive function, sleep, and sex function in Profile 3 may be due to long-term ART (especially integrase inhibitors). For cognitive symptoms, the findings are inconclusive regarding whether ART improves or increases cognitive impairment in PWH^40,41^. Gao and colleagues conducted a systematic review and found that ART did not improve cognitive function in the population of PWH, especially in PWH in moderate and good physical condition^42^. Paul summarized the neurocognitive phenotype of PWH taking ART and found that PWH had poor understanding and reasoning but no loss of memory after receiving ART^43^. The results of these two studies were in line with our results that ART-related cognitive impairment commonly manifested as difficulty in reasoning and understanding in PWH with moderate symptom severity. In the post-ART era, PWH still commonly show evidence of having mild cognitive impairment, which is a significant risk factor for dementia and accelerated ageing^44,45^.

HIV infection and nonnucleoside analogs (e.g., efavirenz) may increase cortisol levels and result in wakefulness, shallow sleep, or decreased REM sleep at night^46,47^. Previous studies reported that HIV-related sleep disorder was closely related to a shorter time since HIV/AIDS diagnosis and ART use. In our study, the time since HIV/AIDS diagnosis and the duration of ART use in PWH in Profile 3 were shorter than those of their counterparts, which could make them more vulnerable to sleep disorders. Moreover, previous studies suggested a potential correlation between sleep disorders and cognitive impairment in PWH^48,49^. However, according to the symptom network of Profile 3 in Fig. 2, we did not find a close relationship between sleep disorders and cognitive impairment. Our results indicated that the relationships may vary in subgroups of the population. For PWH without opportunistic infections and comorbidities and in moderate physical condition, sleep quality may not be associated with the level of cognitive impairment.

### Profile 4: medium symptom severity in the transitional period

Profile 4 was named the “transitional period” and described a subgroup of PWH who recovered from opportunistic infections and comorbidities and transitioned to the “all low” status. For PWH in this profile, their symptom network was sparse, and only symptoms within the clusters were highly correlated. Unlike Profile 2, cognitive symptoms, such as memory loss, were the core symptoms in Profile 4. This finding indicates that cognitive impairment in the transitional period may be mainly caused by HIV rather than ART side effects. Bandera and colleagues found that the level of cognitive impairment could be an indicator of the scale of HIV replication in the central nervous system when viral load could not be detected in blood^50,51^. HIV in the brain can further infect monocytes and lymphocytes that migrate and produce monocyte chemoattractant protein-1, which could damage the structure of the hippocampus and lead to memory loss. Nonetheless, the concentration of ART in the central nervous system is low owing to the existence of the blood–brain barrier^52,53^. Therefore, it is recommended to use ART regimens with higher central nervous system penetration effectiveness scores, such as regimens with nevirapine, zidovudine, and indinavir/r. In PWH with a long time since HIV-positive diagnosis, it is important to perform cognitive screening during routine follow-up visits.

### Profile 5: all high symptom severity

PWH in profile 5 have the opposite characteristics to the “all low” group. They reported a high level of symptom severity and perceived discrimination and a low level of ADL, medication adherence, quality of life, and self-management capacity. The average years since HIV diagnosis and duration of ART use were over 6 and 5 years, respectively, which should be noted. Long-term ART users who had low levels of medication adherence may contribute to high levels of symptom severity in their survivorships. Current clinical practices mainly focus on newly diagnosed and newly ART-prescribed PWH within 6 months^35,54^. We recommend that ART adherence monitoring should also be applied in PWH with an HIV-positive duration over 5 years.

### Limitations

Despite the many strengths of our study, it has several limitations. First, the cross-sectional design data cannot identify the causality of symptoms. Longitudinal studies are warranted to examine the five profiles found by this study. Second, we did not include PWH diagnosed with severe neurocognitive impairment. Therefore, the severity and centrality of the cognitive dysfunction cluster and five cognitive symptoms might be underestimated. Third, information on the types of comorbidities and ART regimens was not collected in our study. Future studies are needed to identify PWH in whom the types of comorbidities and ART regimens are likely to be categorized into Profile 2 and Profile 3. Fourth, as LPA is a patient-center analytic approach, the results of 5 profiles can only be generalized into similar populations. The convenience method of sampling limits the generalizability of the findings. The conclusion can only be generalized to central, southern, and eastern China in a similar context. Future studies could be conducted in different contexts using different symptom checklists to prove the generalizability of our results. Finally, we used self-reported measures to assess symptoms, medication adherence, quality of life, and self-management capacity. Self-reported assessments may be associated with biases, particularly for psychological symptoms and medication adherence. Future studies are warranted to use objective assessments to strengthen and consolidate our findings.

## Conclusion

The findings of our study identified five profiles in 2927 PWH in China. Our study demonstrates the need for health care professionals to provide PWH with group-specific symptom management interventions based on five profiles to improve their physical and psychological well-being. It is crucial for health care providers to understand that although PWH in profiles 2, 3, and 4 have a moderate level of symptom severity, the potential cause and handling methods are different. Future studies should be conducted in different contexts using different symptom checklists to further validate our results.

## Supplementary Information

Supplementary Information.

## Data Availability

The data will not be shared because the information of people living with HIV/AIDS must be kept confidential.
