# Supplementary material for: Identifying subtypes of HIV/AIDS-related symptoms in China using latent profile analysis and symptom networks
Source: Sci Rep. 2022 Aug 2;12:13271. doi: 10.1038/s41598-022-17720-z (PMC9345945; doi:10.1038/s41598-022-17720-z)
Supplement: Supplementary file 1 — Supplementary Information. [file 41598_2022_17720_MOESM1_ESM.pdf]

```
################################################################
################# General information #########################
################################################################

#RCODE of Yang et al. 2022;
#Title: Identifying subtypes of HIV/AIDS-related symptoms in China using latent profile analysis and symptom networks

#Data are a part of the dataset reported in Yang et al. 2022;
#Mplus 8.1 was used to perform LPA;

################################################################
################################################################

title:
   PLA;
data:
   file is "E:\LPA\LPA.csv";
variable:
   names = C1 C2 C3 C4 C5 S1 S2 S3 S4 S5 S6 S7;
   usevariables = C1 C2 C3 C4 C5 S1 S2 S3 S4 S5 S6 S7;
   classes=c(5);
analysis:
   type=mixture;
PLOT:
   TYPE=PLOT3;
   SERIES IS C1 (1) C2 (2) C3 (3) C4 (4) C5 (5) S1 (6) S2 (7) S3 (8) S4 (9) S5 (10) S6 (11) S7 (12);
model:
OUTPUT:
   TECH11 TECH14;
savedata:
   File is "E:\LPA\LPA.txt";
   Save is cprob;
   Format is free;
```
